# Transdermal deferoxamine administration improves excisional wound healing in chronically irradiated murine skin

**DOI:** 10.1186/s12967-022-03479-4

**Published:** 2022-06-17

**Authors:** Hendrik Lintel, Darren B. Abbas, Christopher V. Lavin, Michelle Griffin, Jason L. Guo, Nicholas Guardino, Andrew Churukian, Geoffrey C. Gurtner, Arash Momeni, Michael T. Longaker, Derrick C. Wan

**Affiliations:** 1 Hagey Laboratory for Pediatric Regenerative Medicine, Stanford University School of Medicine, Stanford, CA USA; 2 Department of Surgery, University of Arizona, Tucson, AZ USA; 3 Institute for Stem Cell Biology and Regenerative Medicine, Stanford University Medical Center, Stanford, CA USA; 4 Department of Surgery, Hagey Family Faculty Scholar in Pediatric Regenerative Medicine, Stanford University School of Medicine, 257 Campus Drive West, Stanford, CA 94305 USA

**Keywords:** Ionizing radiation, Wound healing, Deferoxamine, Mouse model

## Abstract

**Background:**

Radiation-induced skin injury is a well-known risk factor for impaired wound healing. Over time, the deleterious effects of radiation on skin produce a fibrotic, hypovascular dermis poorly suited to wound healing. Despite increasing understanding of the underlying pathophysiology, therapeutic options remain elusive. Deferoxamine (DFO), an iron-chelating drug, has been shown in prior murine studies to ameliorate radiation-induced skin injury as well as improve wound healing outcomes in various pathologic conditions when administered transdermally. In this preclinical study, we evaluated the effects of deferoxamine on wound healing outcomes in chronically irradiated murine skin.

**Methods:**

Wild-type mice received 30 Gy of irradiation to their dorsal skin and were left to develop chronic fibrosis. Stented excisional wounds were created on their dorsal skin. Wound healing outcomes were compared across 4 experimental conditions: DFO patch treatment, vehicle-only patch treatment, untreated irradiated wound, and untreated nonirradiated wounds. Gross closure rate, wound perfusion, scar elasticity, histology, and nitric oxide assays were compared across the conditions.

**Results:**

Relative to vehicle and untreated irradiated wounds, DFO accelerated wound closure and reduced the frequency of healing failure in irradiated wounds. DFO augmented wound perfusion throughout healing and upregulated angiogenesis to levels observed in nonirradiated wounds. Histology revealed DFO increased wound thickness, collagen density, and improved collagen fiber organization to more closely resemble nonirradiated wounds, likely contributing to the observed improved scar elasticity. Lastly, DFO upregulated inducible nitric oxide synthase and increased nitric oxide production in early healing wounds.

**Conclusion:**

Deferoxamine treatment presents a potential therapeutic avenue through which to target impaired wound healing in patients following radiotherapy.

## Introduction

By 2030, it is estimated there will be 4.2 million radiation-treated cancer survivors in the US [1]. Despite being a highly effective therapy for solid tumors, ionizing radiation treatment can be associated with severe acute and chronic adverse effects. Radiation-induced fibrosis (RIF) is a chronic sequelae of the collateral injury to soft tissues in the radiated field that most frequently affects the skin. Radiodermatitis impacts the large majority of patients receiving radiotherapy and can vary from transient skin erythema to ulceration, fistula formation, and skin necrosis [2, 3]. It also presents a major surgical challenge for those needing reconstruction of the irradiated area as major wound healing complications are noted to occur in up to 35% of post-operative patients [4, 5].

Impaired wound healing in irradiated skin is a multifactorial process. Initially, ionizing radiation induces DNA damage and generates free radicals which generate substantial cellular injury, inflammation, and microvascular damage [6, 7]. Over time, inflammation endures secondary to local tissue hypoxia and eventually progresses to chronic fibrosis, a state characterized by a hypocellular and hypovascular dermis bathed in a persistently pro-inflammatory environment [3, 8]. These alterations in the dermal microenvironment present the foundation for compromised wound healing in irradiated tissues [9].

The normal orderly sequence of inflammation, proliferation, and remodeling in wound healing is disrupted by radiation due to a combination of the aforementioned cellular dysfunction, impaired extracellular matrix production, and diminished nutrient delivery. Irradiated fibroblasts and myofibroblasts demonstrate impaired collagen deposition and contractile force generation in the acute wound healing phases, respectively. Reduced keratinocyte proliferation results in delayed epithelial migration and hyperplasia [10]. Granulation tissue formation is also delayed due to impaired collagen deposition resulting in significantly reduced collagen density and weaker breaking strength in the healing wound bed [11]. The slow healing rates and poor quality of healing are further amplified by microvascular insufficiency and decreased angiogenesis causing poor nutrient supply to the wound bed. Though novel agents and experimental treatments, including topical fibronectin, transforming growth factor beta modulators, and stem cell therapy have been investigated, current therapeutic approaches to managing these wound healing complications in irradiated skin are often limited to supportive wound care with an absence of strong literature favoring any particular treatment [12, 13].

In this study, we investigated deferoxamine (DFO) as a potential treatment modality for improving acute wound healing outcomes in chronically irradiated skin. DFO is a Food and Drug Administration (FDA)-approved iron chelating agent which has been shown in prior studies to mitigate radiation-induced chronic skin fibrosis [14, 15]. When applied topically via a reverse micelle delivery vehicle, DFO has also been shown to improve healing outcomes in various types of wounds including diabetic wounds, pressure ulcers and sickle cell ulcers [16–18]. DFO’s therapeutic effects are thought to be mediated through two major mechanisms: (1) a reduction in iron-mediated reactive oxygen species (ROS) generation through its iron scavenging properties resulting in reduced oxidative stress and inflammation and (2) stabilization of hypoxia-inducible factor 1α (HIF1α), a potent transcription factor for numerous pro-angiogenic genes, resulting in improved tissue vascularization. DFO has also been shown to improve collagen deposition and fibril organization in numerous studies [15, 18, 19].

In this preclinical study, we hypothesized that transdermally delivered DFO can improve acute wound healing in chronically irradiated tissue via a reduction in ROS generation, improved tissue vascularity, and improved collagen organization.

## Methods

### Animals and irradiation

**Fig. 1:**
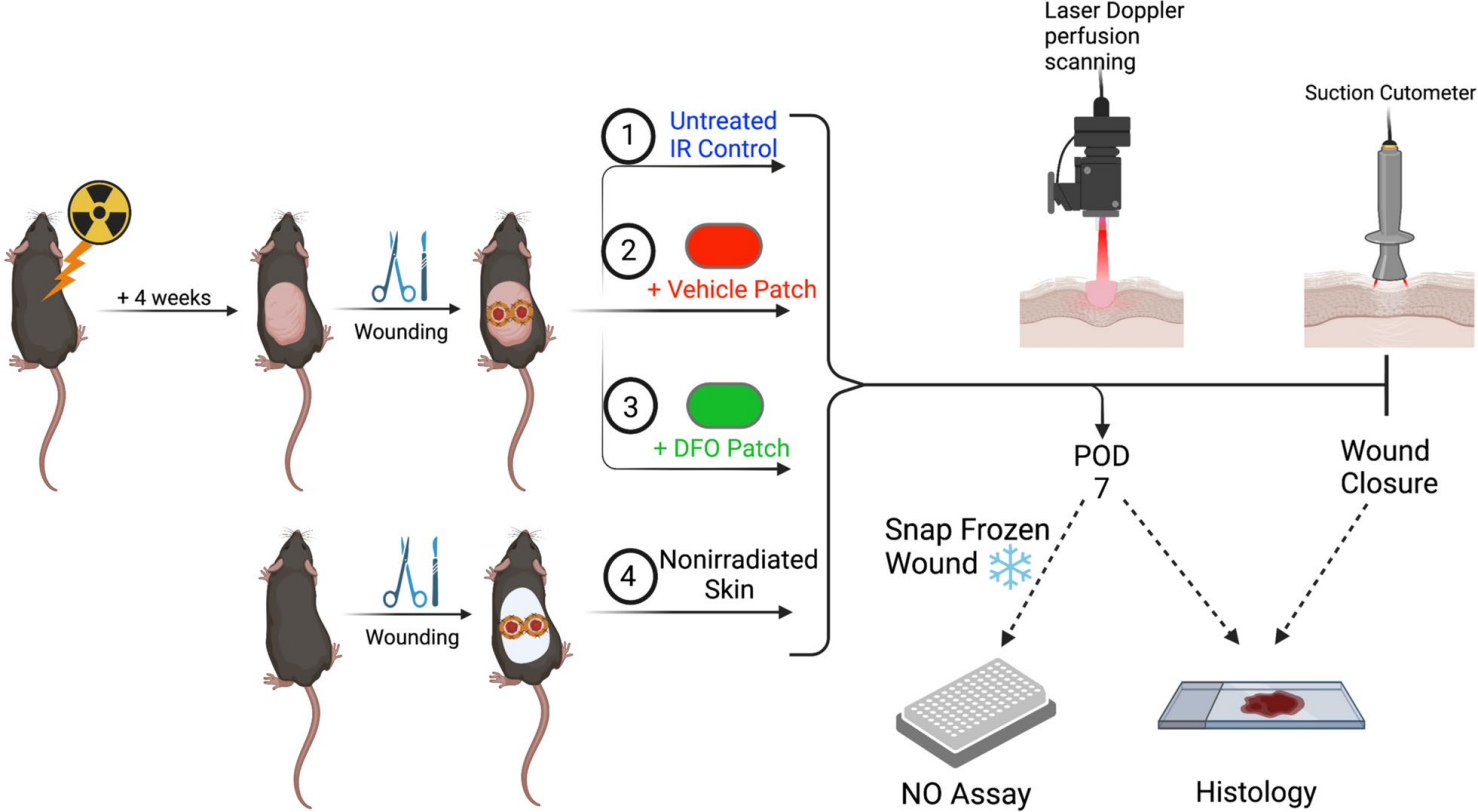
Schematic of mouse allocation across experimental conditions and a timeline of analyses performed on the wounds. Two wounds from each treatment condition were harvested and snap frozen at POD7 for NO assay. IR: irradiation; DFO: deferoxamine; POD: post-operative day; NO: nitric oxide

A total of forty-eight C57BL/6 mice (The Jackson Laboratory, Bar Harbor, ME) were separated into four experimental groups. Thirty six mice were allocated to study irradiated wound healing and divided equally into the 3 treatment conditions: DFO, vehicle-only patch, and untreated control. Prior to irradiation, the dorsal skin was shaved with clippers and treated with Nair™ depilatory cream. Lead shielding was used to protect all tissue except the dorsal skin. The exposed dorsal skin of these mice was irradiated with a total of 30 Gy, fractionated into 6 doses of 5 Gy every other day via a Kimtron Polaris SC-500 X-ray machine (Kimtron Inc., Oxford, CT). Following radiation, mice were left to recover for 4 weeks to allow for the development of chronic fibrosis [14, 15]. Twelve mice were used for control wounds which were created in normal, nonirradiated skin. Two mice from each condition were harvested at post-operative day (POD) 7 for nitric oxide related studies. Allocation of mice and subsequent longitudinal testing are highlighted in Fig. 1. Experiments were approved by the Administrative Panel on Laboratory Animal Care at Stanford University.

### Wounding and treatment

Wounds were created on the dorsal skin in accordance with a well-established silicone-stented excisional wound model [20]. Mice were placed under anesthesia using 2% isoflurane (VetOne, Boise, ID) at a flow rate of 2 L/min. Once sedated, skin was cleaned using alcohol swabs and sterile technique was ensured during wounding. Two excisional wounds of 6 mm diameter were created on each mouse, one on either side of the dorsal mid-line, and stented open with silicone rings. Rings were glued down (Gorilla Glue Co., Cincinnati, OH) then permanently held in place with circumferential 5–0 nylon interrupted suture (Covidien, Dublin, Ireland). For mice receiving patch treatments, patches were placed directly on the open wound. The DFO reverse micelle transdermal delivery patches (TauTona Group, Redwood City, CA) were dosed at 1 mg/cm^2^. Vehicle control patches were the same reverse micelle patch formulation without DFO. The wounded dorsums were dressed with Tegaderm (3 M, Saint Paul, MN). Dressings and patches were changed every two days, until wound closure.

### Perfusion scanning and biomechanical testing

Blood flow in the healing wounds was followed longitudinally via Laser doppler perfusion imaging with a PeriScan PIM 3 (Perimed, Las Vegas, NV) until wound closure. Imaging was performed under inhaled anesthesia and with a constant ambient room temperature every session. Biomechanical testing of each wound was performed upon healing with a Cutometer Dual MPA 580 (Courage + Khazaka Electronic, Cologne, Germany). This provided in vivo measures of scar elasticity via laser-detected skin displacement during suction and release at a negative pressure of 300 millibar with a 2 mm aperture suction probe.

### Tissue harvest

Wound harvest was performed upon wound closure immediately following euthanasia. Irradiated wounds that failed to close by POD21 were harvested the following day and were considered “non-healing”. This determination was based on prior studies and our own pilot wounding studies, which indicated that healing would no longer progress after POD21 as re-epithelialization had occurred but wounds were too fragile, preventing complete healing and full closure [10]. Harvested wounds were fixed in 10% neutral buffered formalin overnight, embedded in paraffin, and then sectioned into 8 μM thick sections. Additionally, two wounds per condition were harvested at POD7 and snap frozen in liquid nitrogen for the nitric oxide assay.

### Histological analysis on healed wounds

Hematoxylin and eosin (H + E), Masson’s Trichrome (TC), and Picrosirius Red (Picro) staining were performed on healed wound sections. Images of each stain were taken on a Leica DMI4000B inverted microscope (Leica Microsystems, Wetzlar, Germany). H + E stains imaged at 10 × were used to assess wound thickness, measuring from epidermis to the base of the granulation tissue (n = 20 per condition). Wound thickness was calculated using the ImageJ (NIH, Bethesda, MD) ruler tool with a scale bar as standard reference. TC stains imaged at 10 × magnification (n = 20 per condition) were used to assess wound collagen density. This was calculated from the density of blue in each image as assessed by the Color Deconvolution 2 plugin for ImageJ [21, 22]. Picro stained specimens, imaged at 40x (n = 200 per condition) with a polarizing lens, were used to examine collagen ultrastructure. Images were analyzed in Matlab (Mathworks, Natick, MA) through a previously described unsupervised machine learning algorithm for the quantification of 294 local and global collagen fiber features including diameter, orientation, maturity, among others [23].

Immunofluorescent staining for CD31 was also performed on healed wound sections. Sections were incubated with anti-CD31 primary antibody (1:100, Abcam ab56299, Cambridge, UK) followed by an Alexa Fluor 594-conjugated donkey anti-rat IgG secondary antibody (1:200; Abcam ab150156). Images taken at 20 × magnification (n = 10 per condition) were used to calculate the red pixel particle count through ImageJ via color thresholding, image binarization, and automated counting of highlighted pixels.

### iNOS staining and nitric oxide assay

Immunofluorescent staining for inducible nitric oxide synthase (iNOS) was performed on wound specimens harvested at POD7. Sections were incubated with anti-iNOS primary antibody (1:50, Abcam ab3523) followed by an Alexa Fluor 488-conjugated goat anti-rabbit IgG secondary antibody (1:200; Invitrogen A11008, Waltham, MA). Images taken at 20 × magnification (n = 12 per condition) were used to calculate the green pixel particle count through ImageJ via color thresholding, image binarization, and counting of highlighted pixels.

Nitric oxide (NO) quantification was performed on the snap frozen wounds harvested at POD7 via a nitric oxide colorimetric assay (Abcam ab65328). Per the manufacturer’s instructions, two wounds from each condition (15 mg each) were suspended in assay buffer and homogenized using a Dounce homogenizer. After centrifuging, the supernatant was isolated, deproteinized and added to a 96-well microplate. Following incubation of the assay fluid in nitrate reductase and the addition of Griess reagents R1 and R2, colorimetric quantification for each wound was performed in triplicate with an Infinite M Nano + plate reader (Tecan, Mannedorf, Switzerland).

## Statistical analysis

Data were analyzed via Prism 9.3.1 (Graphpad Software, San Diego, CA). Difference in the means of the four treatment conditions was determined via one-way analysis of variance (ANOVA). Differences between individual groups were calculated via Tukey’s multiple comparison testing. Statistical significance was determined at a p-value < 0.05. Error bars on graphs in the figures represent 95% confidence intervals.

## Results

### Deferoxamine accelerates wound closure in irradiated skin

**Fig. 2:**
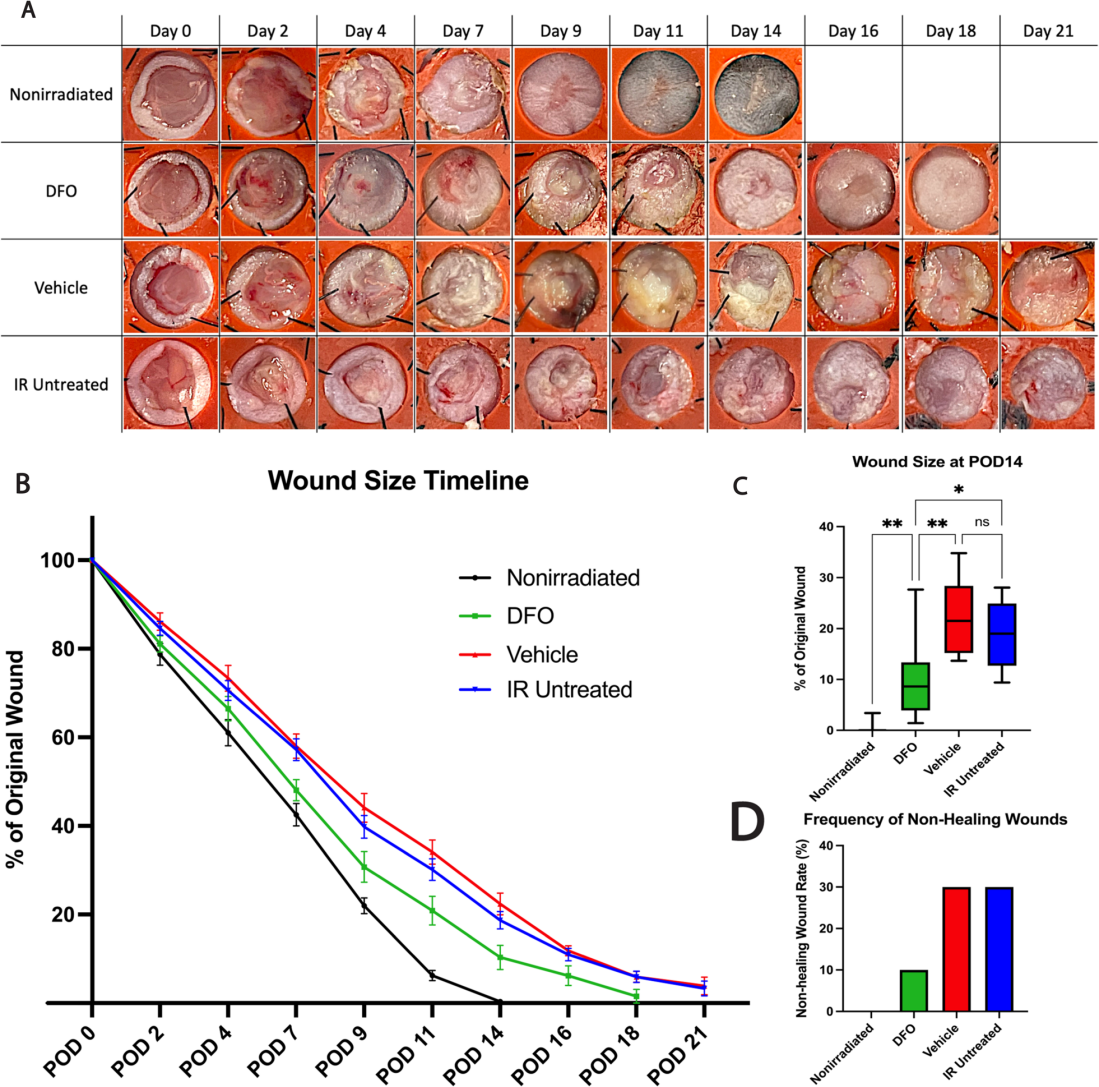
Gross wound healing measures. **A** Representative images of healing progression of excisional wounds by treatment condition. **B** Wound size quantification indicates DFO treated IR wounds showed accelerated wound closure relative to vehicle and IR untreated wounds but slower than nonirradiated wounds (n = 10 per condition). **C** Wound size at POD14 shows statistically significant smaller wounds in DFO treatment (mean 10.34%) than vehicle (22.38%;**p < 0.01) and IR untreated wounds (18.71%;*p < 0.05) while nonirradiated wounds had closed (0.03%;**p < 0.01). **D** Vehicle and IR untreated wounds had a higher rate of non-healing (n = 3) than DFO treated irradiated wounds (n = 1) at POD21. Nonirradiated wounds all closed. DFO: deferoxamine; IR: irradiation; POD: post-operative day; ns: not significant

To determine how deferoxamine would affect wound healing in irradiated skin, excisional wounds were created on irradiated dorsal skin of mice with RIF. Wounds were treated with a DFO-containing patch and gross healing rates were compared to vehicle-only patch wounds, untreated irradiated wounds, and untreated nonirradiated wounds. DFO wounds had a median wound closure time of post-operative day (POD) 18, close to the nonirradiated wound healing time of POD14 and significantly faster than the vehicle and IR untreated wounds which closed on POD21 (Fig. 2A, B). The average area of the original wound remaining open at POD14, when the nonirradiated wounds closed, was 10.34% for DFO versus 22.38% (**p < 0.01) and 18.71% (*p < 0.05) in vehicle and IR untreated wounds, respectively (Fig. 2C). The frequency of non-healing in the irradiated wounds was lower for the DFO group, with only 10% failing to close by POD21 (n = 1 of 10), versus 30% in the both the vehicle and IR untreated wounds (n = 3 of 10). All nonirradiated wounds exhibited complete closure (Fig. 2D).

### Transdermal deferoxamine increases perfusion in wounds in irradiated tissue

**Fig. 3:**
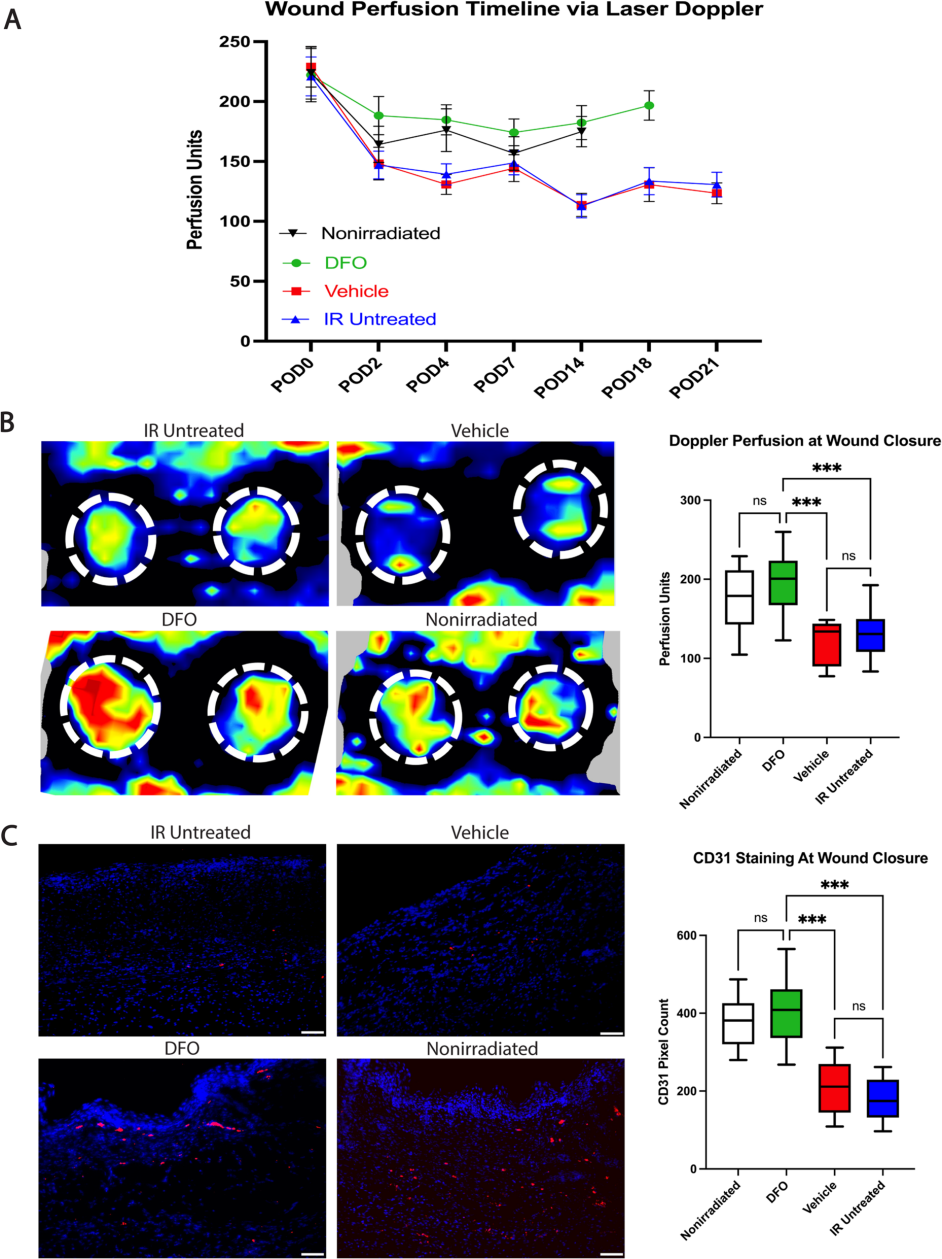
Vascular changes in healing wounds. **A** Perfusion as measured by Laser doppler in wound bed over duration of healing. DFO demonstrates elevated perfusion measures throughout course of healing more closely mimicking nonirradiated wounds relative to radiated controls. **B** Laser Doppler perfusion measures reveal similar perfusion estimates between DFO and nonirradiated wounds when healed (mean: 196.8 vs. 174.9 respectively; p > 0.05). DFO wounds have significantly higher perfusion than vehicle (123.5;***p < 0.001) and IR untreated wounds (130.7;***p < 0.001). Representative images of Doppler scans of healed wounds are shown. **C** CD31 immunofluorescence reveals higher staining of CD31 with DFO (mean 407.4) relative to vehicle (208.0; ***p < 0.001) and IR untreated wounds (179.8; ***p < 0.001), with levels similar to nonirradiated wounds (378.1;p > 0.05). Representative images of CD31 staining on healed wound sections at 20 × magnification depicted per condition. Scale bar = 50 μm. DFO: deferoxamine; IR: irradiation; ns: not significant; POD: post-operative day

Having demonstrated accelerated wound closure in the DFO treated wounds, we evaluated Laser doppler perfusion throughout the course of healing. Notably, DFO-treated wounds mimicked the perfusion index of nonirradiated wounds throughout the healing process, both of which demonstrated elevated perfusion relative to the vehicle and IR untreated wounds at each time point (Fig. 3A). Furthermore, perfusion measures at wound closure, despite different healing times, revealed DFO treated wounds had a mean perfusion index of 196.8, significantly higher than both the vehicle wounds (mean 123.5; ***p < 0.001) and IR untreated wounds (130.7; ***p < 0.001) (Fig. 3B).

Given the perfusion findings, we performed immunohistochemical staining for CD31, an endothelial cell marker, to further explore the elevated perfusion index observed in DFO wounds. Paralleling Laser Doppler results, the irradiated wounds treated with DFO exhibited increased CD31 staining most closely resembling that of the nonirradiated wounds. Of the irradiated wounds, DFO treatment resulted in significantly higher CD31 staining (mean 407.4) than the vehicle wounds (208.0; ***p < 0.001) and IR untreated wounds (179.8; ***p < 0.001) (Fig. 3C).

### Deferoxamine alters collagen composition of healing irradiated wounds

**Fig. 4:**
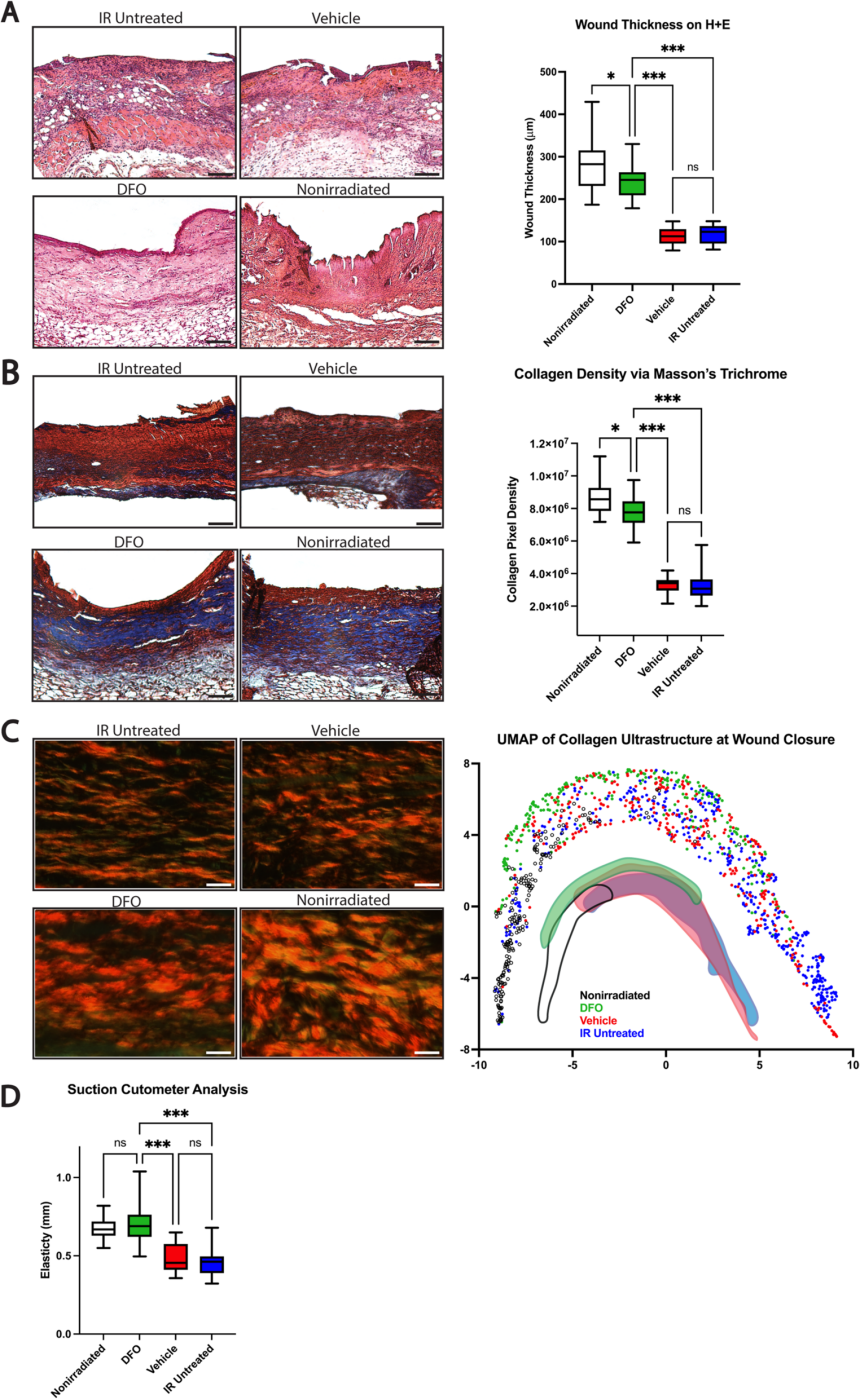
Histologic and suction cutometer analysis of healed wounds. **A** H + E staining of healed specimens revealed DFO treated irradiated wounds resembled nonirradiated wounds more closely in regards to wound thickness (mean 244.6 vs. 284.3 μm, respectively; *p < 0.05) rather than much thinner vehicle (111.7; ***p < 0.001) and IR untreated wounds (116.5; ***p < 0.001). Representative H + E images of healed wounds of each treatment condition taken at 10 × magnification shown (left). **B** On TC staining, DFO wounds also had similar collagen density (mean 7.79 × 10^6^) to nonirradiated wounds (8.69 × 10^6^; *p < 0.05) after healing, both of which had significantly higher density than vehicle (3.31 × 10^6^; ***p < 0.001) and IR untreated wounds (3.24 × 10^6^; ***p < 0.001). Representative TC images of healed wounds of each treatment condition taken at 10 × magnification shown (left). **C** Representative appearance of collagen in Picro images taken at 40 × magnification (left). UMAP representation of collagen ultrastructure in wounds in each treatment condition (right). An approximation of the clustering pattern beneath plotted points highlights DFO (green) clustering more closely to nonirradiated wounds (clear) than vehicle (red) or IR untreated wounds (blue). **D** Elasticity measured as skin displacement upon application of fixed negative pressure indicated healed DFO wounds had similar elasticity to nonirradiated wounds (mean 0.695 vs. 0.675 mm; p > 0.05). Healed vehicle (0.479;***p < 0.001) and IR untreated (0.465;***p < 0.001) wounds were found to be more stiff. Black scale bar = 100 μm. White scale bar = 25 μm. DFO: deferoxamine; H + E: Hematoxylin and Eosin; IR: irradiation; mm: millimeter; ns: not significant; Picro: Picrosirius Red; POD: post-operative day; TC: Masson’s Trichrome; UMAP: Uniform Manifold Approximation and Projection

In addition to assessing the rate of wound closure and its underlying drivers, we also sought to characterize the effect of deferoxamine therapy on the quality of wound healing. Wound thickness assessed via H + E revealed deferoxamine increased wound thickness (mean 244.6 μm) compared to the vehicle (111.7; ***p < 0.001) and IR untreated wounds (116.5; ***p < 0.001). Interestingly, DFO restored wound thickness in irradiated skin to a similar level to that observed in the wounds of nonirradiated skin (284.3; *p < 0.05) (Fig. 4A). Wound collagen density, assessed via Masson’s trichrome staining, similarly revealed increased collagen density in the DFO wounds (mean 7.79 × 10^6^) versus those of the vehicle (3.31 × 10^6^; ***p < 0.001) and IR untreated group (3.24 × 10^6^; ***p < 0.001) (Fig. 4B). The collagen density of irradiated wounds treated with DFO more closely resembled the density seen in the nonirradiated wounds (8.69 × 10^6^; *p < 0.05) than the irradiated conditions, mirroring the H + E wound findings (Fig. 4A, B). Importantly, these histological findings were limited directly to the wound and were not observed in surrounding irradiated, uninjured skin which demonstrated increased dermal thickness and collagen density secondary to chronic RIF.

Picrosirius Red staining was used to assess the collagen ultrastructure of the healed wounds. When analyzed through a supervised machine learning algorithm, DFO-treated wounds were found to cluster distinctly from the vehicle and IR untreated wounds, and were closer in ultrastructure to the nonirradiated wounds, displayed by their proximity on a uniform manifold approximation and projection (UMAP) (Fig. 4C). The histologic findings regarding collagen deposition and organization were substantiated mechanically through assessment of wound elasticity via suction cutometer testing. DFO-treated irradiated wounds (mean 0.695 mm) and nonirradiated wounds (0.675; p > 0.05) demonstrated similar scar elasticity, and both were found to be significantly more elastic than the vehicle (0.479;***p < 0.001) and IR untreated (0.465;***p < 0.001) scar tissue (Fig. 4D).

### Deferoxamine augments iNOS expression in irradiated wounds

**Fig. 5:**
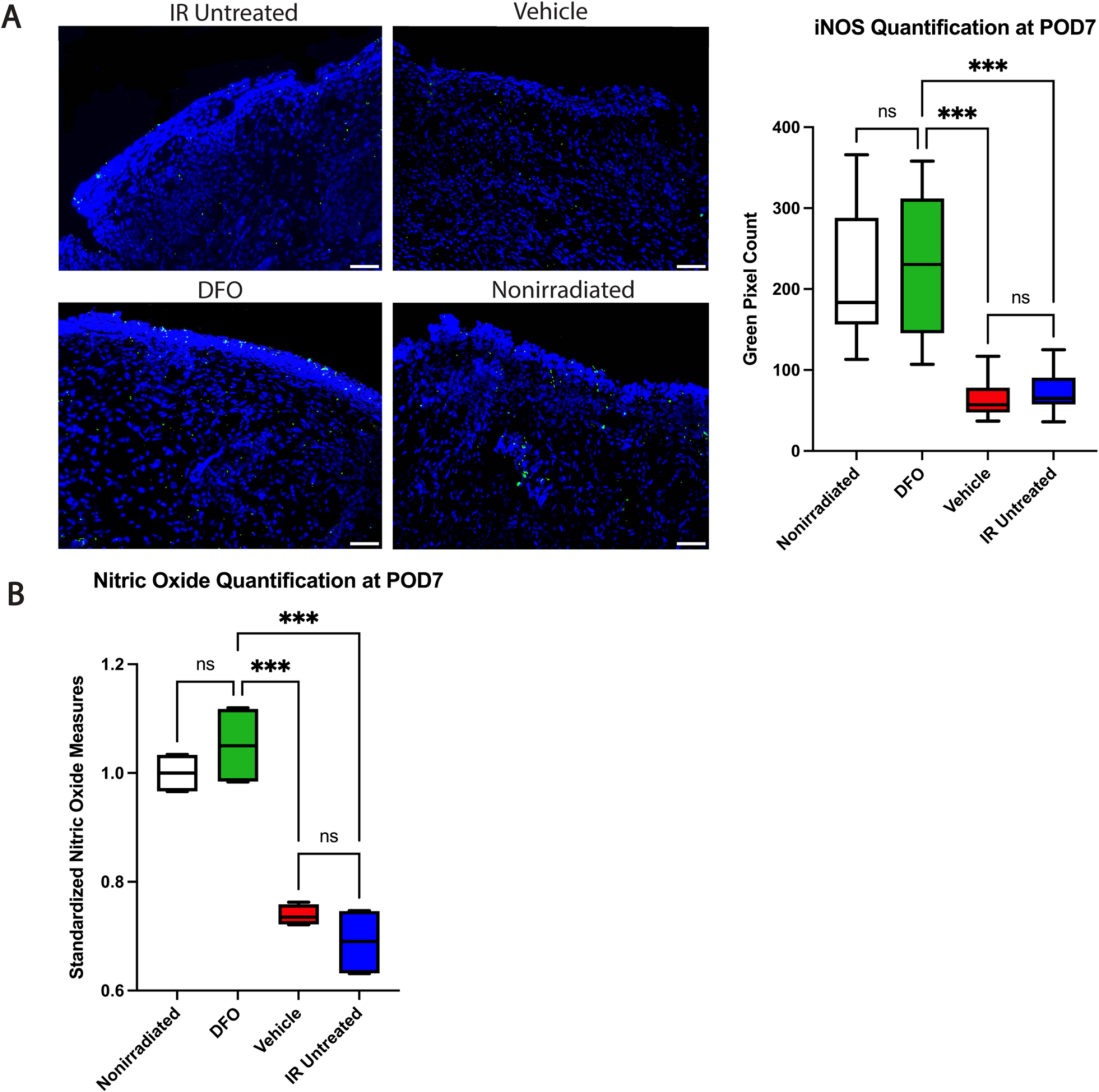
Nitric oxide in healing irradiated wounds at POD7. **A** Representative images of iNOS staining on wound sections taken at 20 × magnification depicted per condition (left). iNOS staining revealed increased iNOS in DFO-treated wounds (mean 229.1) relative to that observed in vehicle (64.3;***p < 0.001) and IR untreated wounds (71.0;***p < 0.001). DFO iNOS quantification was comparable to nonirradiated wounds (210.4; p > 0.05). **B** Nitric oxide colorimetric assay normalized to nonirradiated wounds revealed DFO wounds had similar nitric oxide levels (1.051) in wounds at POD7 compared to nonirradiated wounds (1.00; p > 0.05). NO was lower in vehicle (0.74;***p < 0.001) and control (0.69;***p < 0.001) irradiated wounds relative to DFO treated nonirradiated wounds. Scale bar = 50 μm. iNOS: inducible nitric oxide synthase; IR: irradiation; DFO: deferoxamine; POD: post-operative day; NO: nitric oxide; ns: not significant

In order to further explore the increased collagen deposition observed in DFO treated irradiated wounds, the effect of DFO on iNOS activity in the healing wounds was assessed, since nitric oxide is a known regulator of collagen deposition during wound healing. Immunofluorescent staining for iNOS in the wound bed revealed increased iNOS in DFO-treated wounds (mean 229.1) relative to that observed in vehicle (64.3;***p < 0.001) and IR untreated wounds (71.0;***p < 0.001) (Fig. 5A). These findings are further supported through a nitric oxide colorimetric ELISA which revealed increased nitric oxide present in the DFO-treated wounds. Deferoxamine treated irradiated wounds revealed increased NO levels (1.051) resembling that seen in nonirradiated wounds (1.00; p > 0.05), and significantly higher than the levels observed in vehicle (0.74;***p < 0.001) and IR untreated wounds (0.69;***p < 0.001) (Fig. 5B).

## Discussion

Wound healing complications are a well-known consequence of radiation treatment and can present a severe burden for patients who have undergone radiotherapy. The deleterious effects of ionizing radiation on the wound healing cascade are a significant cause of morbidity. Wounds in irradiated tissue suffer from a high rate of adverse effects including failure to heal, infection, the need for debridement of nonviable tissue, and exposure of underlying vital structures [13, 24, 25]. Furthermore, conventional wound care is less effective for irradiated wounds and surgical intervention can often be the only definitive treatment [13, 26]. Given the hypovascular character of irradiated skin, graft failure is a significant concern and often pushes the surgeon to more challenging reconstructions necessitating the use of well-vascularized distal flaps, but even these are at higher risk of failure [27, 28].

Radiation injury is mediated at the cellular level through immediate DNA damage and perpetual free radical production [6, 7]. Over time, this progresses to RIF and leads to the many hurdles impairing tissue healing [3, 8, 9]. In the skin, irradiated fibroblasts and myofibroblasts exhibit altered collagen deposition and reduced wound contraction, respectively [11, 29, 30]. The initial microvascular damage and the subsequent chronic hypovascularity impair nutrient delivery to the healing wound, further delaying the healing process. In spite of the increased research and understanding of the pathophysiology leading to radiation injury, therapeutic options have remained elusive.

In this study, we evaluated transdermal deferoxamine as a potential treatment for improving wound healing outcomes in chronically irradiated skin. Its therapeutic mechanisms directly address many of the pathophysiologic processes underlying impaired healing following radiation injury. We hypothesized that the reduced oxidative stress, through DFO’s iron scavenging properties, and its ability to upregulate angiogenesis, through the stabilization of HIF1α, would lead to a favorable microenvironment for improved wound healing outcomes. To this end, we investigated gross wound closure rates, histological appearance of healed tissue, wound perfusion throughout healing, and scar elasticity in irradiated wounds treated with DFO compared to irradiated and nonirradiated controls.

### Deferoxamine improves wound healing rate and reduces non-healing wound frequency in chronic irradiated skin

Using a well-established murine excisional wound model, we demonstrated that DFO accelerates the closure of acute wounds made in chronically irradiated skin. Prior murine studies have demonstrated similar effects of deferoxamine on various wounds including diabetic ulcers, pressure wounds, and sickle-cell ulcers [16–18]. Furthermore, compared to untreated irradiated wounds, DFO also reduced the frequency of non-healing. The observed failure to close in some of our irradiated wounds is reminiscent of clinical observations noted by plastic surgeons during operations performed in irradiated fields. Some studies suggest up to 35% of patients with pre-operative radiation therapy suffer from wound closure complications [5, 31]. The importance of accelerated wound closure is self-evident as it reduces the risk for infection and minimizes exposure of underlying vital structures to the nonsterile outer world [32].

### Deferoxamine improves wound perfusion and angiogenesis

Laser doppler perfusion scanning performed over the course of wound healing revealed DFO significantly improved blood flow in irradiated wounds, preserving perfusion to a degree seen in nonirradiated wounds, which is significant as the initial wound occurs in an already hypovascular field. Through its modulation of the degradation pathway of HIF1α, DFO triggers the upregulation of angiogenic transcription factors, such as vascular endothelial growth factor (VEGF), and increases angiogenesis–a therapeutic effect which has been demonstrated previously in murine models of RIF and other excisional wounds [15, 16, 33, 34]. CD31 staining provided further evidence that DFO also functioned to potentiate angiogenesis in our wound tissue samples. This is notable as dermal hypovascularity, a hallmark of RIF, is responsible for reduced nutrient supply and is likely a key contributor to the impaired wound healing seen in irradiated skin [35]. Prior murine studies have demonstrated a similar correlation between increased angiogenesis and improved healing outcomes in irradiated wounds [10, 12]. Clinically, good perfusion is a sine qua non for wound healing. Routine debridements to restore wound margins with healthy blood flow, the application of nitroglyerin paste, and meticulous doppler follow-up to assess flap viability are just illustrative examples of the importance of this notion in surgical practice.

### Deferoxamine alters collagen deposition in irradiated wounds.

Histological analysis further elucidated the effects of DFO on the wound healing process. DFO treated irradiated wounds were demonstrably thicker than their untreated counterparts, indicative of both increased granulation tissue and a thicker neoepidermis which more closely mimicked that of the nonirradiated wounds. This is in contrast to the untreated irradiated wounds which were much thinner and are known to suffer from reduced granulation tissue formation and a thinner neoepidermis in other studies as well [11]. Both of these factors likely contribute to the clinically well-documented fragility and unpredictability of irradiated wounds [11, 36].

The mechanical fragility is certainly also attributable to both impaired collagen deposition and organization. DFO affected both of these measures. Trichrome staining demonstrated significantly reduced collagen density in the irradiated wounds relative to nonirradiated wounds. DFO treatment increased wound collagen content to that observed in nonirradiated wounds. Low collagen density is a known risk factor for skin fragility [37]. Furthermore, Picrosirius Red staining indicated that DFO also influenced the collagen fiber ultrastructure in the healed wound. An unsupervised machine learning algorithm determined that DFO-treated irradiated wounds more closely resembled nonirradiated wounds based on 294 learned parameters that were examined within the collagen ultrastructure. Prior studies have also asserted DFO’s ability to improve fibril organization in irradiated skin; irradiated wounds typically exhibit thin, disorganized collagen fiber deposition [11, 19]. These differences resulted in improved scar elasticity in the DFO treated irradiated wounds that again aligned more closely to nonirradiated wounds. A more elastic scar provides more mobility and better tension offloading ability, maximizing tolerated tensile strain, thus reducing the risk of dehiscence [38]. In all, these factors each played a significant role in the acceleration of wound closure and reduced frequency of non-healing noted in the DFO treated wounds.

### DFO potentiates iNOS activity in healing irradiated wounds

Given the elevated collagen deposition observed with DFO treatment in this experiment, we sought to test the hypothesis that DFO may exert this effect through the modulation of iNOS activity in the wound. Nitric oxide is known to induce collagen deposition in wounds [39–41]. During wound healing, it is largely produced by iNOS, expressed by both fibroblasts and macrophages in the wound bed [42]. As expected, impaired wound healing and reduced collagen deposition has been well documented in iNOS deficient mice [43–45]. Wounds made in irradiated skin have also been shown to have reduced iNOS expression and produce less nitric oxide [9, 46]. Furthermore, prior in vitro studies have demonstrated that iNOS expression is responsive to HIF1α as the iNOS gene promoter has a hypoxia responsive element [47, 48]. Separate studies have also indicated DFO is able to upregulate iNOS expression via its modulation of HIF1α pathway [49]. In our study, we indeed observed elevated expression of iNOS in the DFO-treated irradiated wounds relative to the untreated irradiated wounds. Nitric oxide levels were also found to be elevated in the DFO treated wounds relative to the untreated irradiated wounds. These findings suggest that the iNOS pathway may be one of the mechanisms through which DFO exerts its effect on increasing collagen deposition in healing irradiated wounds.

## Limitations

Although wound healing research has come a long way, murine models remain an imperfect proxy for human wound healing. Despite the use of a stented excisional wound model which maximizes the contribution of re-epithelialization and granulation tissue formation to healing, wound closure can still marginally be affected by contraction of the panniculus carnosus [50]. Furthermore, although we used well-established methods for inducing chronic fibrosis, murine RIF is still considered an approximation of RIF in human skin due to differences in skin layer thicknesses and structure [51]. Lastly, we established that irradiated wounds treated with DFO exhibit elevated NO during healing. We believe this is largely attributable to the increased iNOS expression we demonstrated via immunofluorescence given that this is the predominant NOS isoform present in early healing wounds [42]. However, given DFO’s vasculogenic properties, elevated levels of endothelium-derived nitric oxide via endothelial NOS (eNOS) could also have contributed to some increased NO production during healing [52, 53]. Regardless of the degree of eNOS contribution, we noted a functional increase in nitric oxide during wound healing secondary to DFO administration which may have contributed to the improved healing outcomes we observed.

## Conclusion

Transdermally administered deferoxamine accelerated and improved wound healing of excisional wounds made on chronically irradiated murine skin. DFO augmented angiogenesis leading to increased blood flow to the healing wound bed. DFO also increased collagen deposition and organization in the healed wound, leading to a wound more closely resembling a nonirradiated wound histologically and biomechanically on scar elasticity testing. Lastly, DFO also demonstrated an ability to potentiate iNOS activity in the healing wound bed, increasing nitric oxide production and leading to elevated collagen deposition and improved healing. These findings indicate deferoxamine has strong potential as a therapeutic option for patients suffering from impaired wound healing in irradiated skin.

## Data Availability

The datasets used and/or analyzed during the study are available from the corresponding author on reasonable request.

## Abbreviations

DFO: Deferoxamine
eNOS: Endothelial nitric oxide synthase
FDA: Food and Drug Administration
H + E: Hematoxylin and Eosin
HIF1α: Hypoxia-inducible factor 1α
IR: Irradiation
mm: Millimeter
NO: Nitric oxide
iNOS: Inducible nitric oxide synthase
ns: Not significant
Picro: Picrosirius Red
POD: Post-operative day
RIF: Radiation induced fibrosis
ROS: Reactive oxygen species
TC: Masson’s Trichrome
UMAP: Uniform Manifold Approximation and Projection
